# Assessing the diagnostic utility of the Gaucher Earlier Diagnosis Consensus (GED-C) scoring system using real-world data

**DOI:** 10.1186/s13023-024-03042-y

**Published:** 2024-02-16

**Authors:** Shoshana Revel-Vilk, Varda Shalev, Aidan Gill, Ora Paltiel, Orly Manor, Avraham Tenenbaum, Liat Azani, Gabriel Chodick

**Affiliations:** 1 Gaucher Unit, Shaare Zedek Medical Center, Jerusalem, Israel; 2 Faculty of Medicine, Hebrew University, Jerusalem, Israel; 3 Braun School of Public Health and Community Medicine, Hebrew University, Jerusalem, Israel; 4 Sackler School of Medicine, Tel Aviv University, Tel Aviv, Israel; 5 Takeda Pharmaceuticals International AG, Zurich, Switzerland; 6 Department of Hematology , Hadassah Medical Organization, Jerusalem, Israel; 7 MaccabiTech, Maccabi Healthcare Services, Tel Aviv, Israel

**Keywords:** Gaucher disease, Algorithm, Early diagnosis, Real-world data, Gaucher earlier diagnosis consensus scoring system

## Abstract

**Background:**

Gaucher disease (GD) is a rare autosomal recessive condition associated with clinical features such as splenomegaly, hepatomegaly, anemia, thrombocytopenia, and bone abnormalities. Three clinical forms of GD have been defined based on the absence (type 1, GD1) or presence (types 2 and 3) of neurological signs. Early diagnosis can reduce the likelihood of severe, often irreversible complications. The aim of this study was to validate the ability of factors from the Gaucher Earlier Diagnosis Consensus (GED-C) scoring system to discriminate between patients with GD1 and controls using real-world data from electronic patient medical records from Maccabi Healthcare Services, Israel’s second-largest state-mandated healthcare provider.

**Methods:**

We applied the GED-C scoring system to 265 confirmed cases of GD and 3445 non-GD controls matched for year of birth, sex, and socioeconomic status identified from 1998 to 2022. The analyses were based on two databases: (1) all available data and (2) all data except free-text notes. Features from the GED-C scoring system applicable to GD1 were extracted for each individual. Patients and controls were compared for the proportion of the specific features and overall GED-C scores. Decision tree and random forest models were trained to identify the main features distinguishing GD from non-GD controls.

**Results:**

The GED-C scoring distinguished individuals with GD from controls using both databases. Decision tree models for the databases showed good accuracy (0.96 [95% CI 0.95–0.97] for Database 1; 0.95 [95% CI 0.94–0.96] for Database 2), high specificity (0.99 [95% CI 0.99–1]) for Database 1; 1.0 [95% CI 0.99–1] for Database 2), but relatively low sensitivity (0.53 [95% CI 0.46–0.59] for Database 1; 0.32 [95% CI 0.25–0.38]) for Database 2). The clinical features of splenomegaly, thrombocytopenia (< 50 × 10^9^/L), and hyperferritinemia (300–1000 ng/mL) were found to be the three most accurate classifiers of GD in both databases.

**Conclusion:**

In this analysis of real-world patient data, certain individual features of the GED-C score discriminate more successfully between patients with GD and controls than the overall score. An enhanced diagnostic model may lead to earlier, reliable diagnoses of Gaucher disease, aiming to minimize the severe complications associated with this disease.

## Background

Gaucher disease (GD) is a rare autosomal recessive condition characterized by a deficiency of the lysosomal enzyme beta-glucocerebrosidase (*GBA1*). Accumulation of glucosylceramide in macrophages throughout the body leads to the onset of multisystemic disease manifestations such as splenomegaly, hepatomegaly, anemia, thrombocytopenia, and bone abnormalities, hallmarks of type 1 GD [1]; neurological involvement is characteristic of the more severe type 2 and type 3 GD [2, 3]. The estimated prevalence of all three GD types is 0.45–25.0 per 100,000 live births, although type 1 GD is substantially more common among people with Ashkenazi Jewish heritage (estimated at 1 in 850 live births for type 1 GD) [4, 5].

Timely initiation of appropriate GD-specific therapy (i.e., enzyme replacement therapy or substrate reduction therapy) early in the disease course [6–8] has been shown to improve patient outcomes, with significant effects on hematologic and visceral outcomes, and may prevent the onset of irreversible bone disease and severe growth retardation, and reduce the risk of bleeding [8]. However, delayed diagnosis or misdiagnosis is frequent, owing to the complex and non-specific clinical presentation, together with a lack of awareness about this rare disease [1, 9–11]. Approximately one in six patients report remaining undiagnosed for 7 years or more after first consulting a doctor with symptoms [9]. Physicians and patients both report multiple referrals to a range of different specialties prior to GD diagnosis, with primary care, hematology/hematology-oncology, and pediatrics being the main specialties to which patients first present symptoms [9]. 

The Gaucher Earlier Diagnosis Consensus (GED-C) scoring system was developed by a panel of 22 expert physicians using Delphi methodology regarding the signs and covariables considered important for diagnosing type 1 and type 3 GD, to help clinicians identify potential individuals to test further, thereby reducing diagnostic delay [12]. Preliminary validation of the GED-C was able to discriminate between patients with GD and those with overlapping manifestations from other disorders, in studies from the United Kingdom [13] and Finland [14]. We previously carried out a description of the GED-C scoring system in 265 confirmed patients with GD using real-world data from the Maccabi Healthcare Services (MHS), Israel’s second-largest state-mandated healthcare provider, representing 2.5 million members (25% of the Israeli population) [15]. The aim of the current study was to assess the ability of the GED-C score to discriminate between individuals with and without GD, and to identify the best discriminatory features using real-world data from electronic patient medical records from the MHS, with and without the use of free-text notes.

## Methods

### Data source and study design

Electronic patient medical records from the MHS were used as the data source for this study. In the MHS, clinical records have been fully computerized for > 20 years, and are fully integrated with an automated central laboratory, fully digitized imaging, and pharmacy purchase data. In addition, the MHS is associated with a biobank of samples collected from consenting sample donors among MHS participants. The study design was approved by the MHS Institutional Review Board (0013-21-ASMC). The requirement for patient consent was waived owing to the use of de-identified, anonymized data.

### Population

All eligible individuals with a confirmed diagnosis of GD in the MHS database were included in the study, as described previously [15]. The records of patients identified with an MHS diagnosis code for GD were screened for evidence of GD-specific treatment authorization such as enzyme replacement therapy or substrate reduction therapy. In the absence of evidence of GD treatment, patient records were screened for any medical notes indicating GD (e.g., physicians’ notes and hospital discharges). Thirteen randomly selected controls per case, matched for year of birth, sex, and socioeconomic status (per MHS data), were extracted from the MHS database for retrospective analysis.

### Data extraction

Data for each of the GED-C items applicable to type 1 GD were extracted for each individual, including demographics, diagnosis codes (International Classification of Diseases [ICD], Ninth Revision [ICD-9]), laboratory values, imaging reports, the MHS osteoporosis register, and weight and height measurements. Free-text notes were screened manually for the terms presented in Table 1 for all patients with GD and non-GD controls. For the non-GD controls, free-text notes were screened manually in 1 control from the 13 controls available for each patient with GD (chosen randomly). We assumed that the proportion of the terms in the 12 matched controls was similar to the control that was checked manually. Data were extracted from the first record available until 1 year after GD diagnosis. For controls, data were extracted up to 1 year after the GD diagnosis of the matched patient. The 1-year post-diagnosis cutoff was chosen to allow the maximum time for the capture of features on completion of relevant confirmatory testing for GD. Items for GED-C scores were extracted as quantitative values where possible. GED-C scores were calculated for these features, as indicated in Table 2 for all available data, including free text-notes from patient visits (database 1) and Table 3, data from free-text notes from patient visits were not included (database 2).

**Table 1:** GED-C score parameters extracted from free-text notes from patient visits

	GED-C parameters						
	Splenomegaly	Hepatomegaly	Gallstones	Pain	Bleeding	Fatigue	Growth retardation
Patients with GD, n (%) (n = 265)	128 (48.3)	48 (18.1)	19 (7.2)	133 (50.2)	58 (21.9)	75 (28.3)	12 (4.5)
Controls, n (%) (n = 265)^a^	2 (0.8)	6 (2.3)	4 (1.5)	58 (21.9)	24 (9.1)	21 (7.9)	1 (0.4)
OR (95% CI)	122.8 (30.0–504.0)	9.5 (4.0–22.7)	5 (1.7–15)	3.9 (2.7–5.7)	2.8 (1.7–4.7)	4.6 (2.7–7.7)	12.5 (1.6–97.0)

*GD* Gaucher disease; *GED-C* Gaucher earlier diagnosis consensus, *OR* odds ratio

^a^Free-text notes were screened manually in 1 control from the 13 controls available for each patient with GD (chosen randomly)

**Table 2:** Characteristics used to calculate GED-C scores in patients with GD and control patients: database 1

Weighting	Characteristic	Patients with GD, n (%) n = 265	Controls, n (%) n = 3445	OR (95% CI)
3 points	Splenomegaly	135 (50.9)	44 (1.3)	80.3 (54.7–117.7)
2 points	Thrombocytopenia (platelet count 50–150 × 10^9^/L)	107 (60.5) [177 samples]	175 (10.0) [1750 samples]	13.7 (9.8–19.3)
	Bone issues	150 (56.6)	1564 (45.4)	1.6 (1.2–2.0)
	Anemia (Hb 9.5–14.0 g/dL)	147 (83.0) [177 samples]	1362 (77.8) [1750 samples]	1.4 (0.9–2.1)
	Hyperferritinemia (ferritin 300–1000 ng/mL)	45 (41.7) [108 samples]	20 (2.4) [829 samples]	28.9 (16.1–51.9)
	Gammopathy (IgG, IgM, IgA, and IgE)^a^ (> normal range)	29 (40.8) [71 samples]	56 (16.4) [342 samples]	3.5 (2.0–6.1)
	Jewish ancestry	257 (98.1) [262 samples]	3140 (92.4) [3397 samples]	4.5 (1.8–11.0)
1.5 points	Hepatomegaly^b^	52 (19.6)	90 (2.6)	9.1 (6.3–13.1)
1 point	Thrombocytopenia (platelet count < 50 × 10^9^/L)	11 (6.2) [177 samples]	2 (0.11) [1750 samples]	57.9 (12.7–263.0)
	Anemia (Hb < 9.5 g/dL)	16 (9.0) [177 samples]	50 (2.8) [1750 samples]	3.4 (1.9–6.0)
	Hyperferritinemia (ferritin > 1000 ng/mL)	4 (3.8) [104 samples]	1 (0.1) [829 samples]	33.1 (3.7–299.0)
0.5 points	Gallstones	28 (10.6)	119 (3.5)	3.3 (2.1–5.1)
	Bleeding	69 (26.0)	563 (16.3)	1.8 (1.4–2.4)
	Leukopenia (WBC < normal range)	26 (14.7) [177 samples]	82 (4.7) [1750 samples]	3.5 (2.2–5.6)
	Low bone marrow density	64 (24.2)	242 (7.0)	3.5 (2.6–4.7)
	Growth retardation^c^	124 (46.8)	918 (26.6)	2.4 (1.9–3.1)
	Fatigue^d^	85 (32.0)	633 (18.4)	2.1 (1.6–2.7)
	Dyslipidemia (HDL cholesterol < 35 mg/dL)	81 (61.4) [132 samples]	199 (15.2) [1308 samples]	8.9 (6.0–13.0)
	Elevated ACE (> normal range)	12 (80.0) [15 samples]	3 (27.3) [11 samples]	10.7 (1.7–66.7)
	Age at diagnosis < 18 years	50 (18.9)	685 (19.9)^e^	–

*ACE* angiotensin-converting enzyme, *GD* Gaucher disease, *GED-C* Gaucher early diagnosis consensus, *Hb* hemoglobin, *HDL* high-density lipoprotein, *Ig* immunoglobulin, *OR* odds ratio, *WBC* white blood cell

GED-C items not captured in this analysis: cognitive deficit, cardiovascular calcification, pulmonary infiltrates, death of relative due to fetal hydrops and/or with diagnosis of neonatal sepsis of uncertain etiology (0.5 points); disturbed motor function, myoclonus epilepsy, kyphosis, family history of GD (2 points); disturbed oculomotor function (3 points)

^a^Monoclonal or polyclonal

^b^Combined with mild-moderate hepatomegaly scored as 1.5 points

^c^Including low body weight

^d^Combined with asthenia

^e^For controls, date of diagnosis was derived from the date of diagnosis of the matched patient

**Table 3:** Characteristics used to calculate GED-C scores in patients with GD and control patients: database 2

Weighting	Characteristic	Patients with GD, n (%) (n = 265)	Controls, n (%) (n = 3445)	OR (95% CI)
3 points	Splenomegaly	65 (24.5)	18 (0.52)	61.9 (36.0–106.3)
2 points	Thrombocytopenia (platelet count 50–150 × 10^9^/L)	107 (60.5) [177 samples]	175 (10.0) [1750 samples]	13.7 (9.8–19.3)
	Bone issues	95 (35.8)	1168 (33.9)	1.1 (0.8–1.4)
	Anemia (Hb 9.5–14.0 g/dL)	147 (83.0) [177 samples]	1362 (77.8) [1750 samples]	1.4 (0.9–2.1)
	Hyperferritinemia (ferritin 300–1000 ng/mL)	45 (41.7) [108 samples]	20 (2.4) [829 samples]	28.9 (16.1–51.9)
	Gammopathy (IgG, IgM, IgA, and IgE)^a^ (> normal range)	29 (40.8) [71 samples]	56 (16.4) [342 samples]	3.5 (2.0–6.1)
	Jewish ancestry	257 (98) [262 samples]	3140 (92.4) [3397 samples]	4.5 (1.8–11.0)
1.5 points	Hepatomegaly^b^	23 (8.7)	12 (0.35)	27.2 (13.4–55.3)
1 point	Thrombocytopenia (platelet count < 50 × 10^9^/L)	11 (6.2) [177 samples]	2 (0.11) [1750 samples]	57.9 (12.7–263.0)
	Anemia (Hb < 9.5 g/L)	16 (9.0) [177 samples]	50 (2.8) [1750 samples]	3.4 (1.9–6.0)
	Hyperferritinemia (ferritin > 1000 ng/mL)	4 (3.8) [104 samples]	1 (0.1) [829 samples]	33.1 (3.7–299.0)
0.5 points	Gallstones	18 (6.8)	75 (2.2)	3.3 (1.9–5.6)
	Bleeding	27 (10.2)	308 (8.9)	1.2 (0.8–1.7)
	Leukopenia (WBC < normal range)	26 (14.7) [177 samples]	82 (4.7) [1750 samples]	3.5 (2.2–5.6)
	Low bone marrow density	24 (9.0)	93 (2.7)	3.6 (2.2–5.7)
	Growth retardation^c^	13 (4.9)	73 (2.1)	1.8 (0.9–3.2)
	Fatigue^d^	28 (10.6)	398 (11.6)	0.9 (0.6–1.4)
	Dyslipidemia (HDL cholesterol < 35 mg/dL)	81 (61.4) [132 samples]	199 (15.2) [1308 samples]	8.9 (6.0–13.0)
	Elevated ACE (> normal range)	12 (80.0) [15 samples]	3 (27.3) [11 samples]	10.7 (1.7–66.7)
	Family history of PD	0	0	–
	Age at diagnosis < 18 years	50 (18.9)	685 (19.9)^e^	–

*ACE* angiotensin-converting enzyme, *GD*, Gaucher disease, *GED-C* Gaucher early diagnosis consensus, *Hb* hemoglobin, *HDL* high-density lipoprotein, *Ig* immunoglobulin, *OR* odds ratio, *PD* Parkinson’s disease, *WBC* white blood cell

GED-C items not captured in this analysis: cognitive deficit, cardiovascular calcification, pulmonary infiltrates, death of relative due to fetal hydrops and/or with diagnosis of neonatal sepsis of uncertain etiology (0.5 points); disturbed motor function, myoclonus epilepsy, kyphosis, family history of GD (2 points); disturbed oculomotor function (3 points)

^a^Monoclonal or polyclonal

^b^Combined with mild-moderate hepatomegaly scored as 1.5 points

^c^Including low body weight

^d^Combined with asthenia

^e^For controls, date of diagnosis was derived from the date of diagnosis of the matched patient

### Assessing the GED-C scoring system as a discrimination tool

Individuals with GD were compared with controls using features of the GED-C scoring system applicable to patients with type 1 GD [12, 15], and total points were determined for each group. Computation of the GED-C score was carried out as described previously [15]. Briefly, the weight of each feature was set according to the published score [12]. For laboratory data, the maximum or minimum levels (as appropriate) were considered and defined abnormal as indicated in Tables 2 and 3. Dichotomous variables were coded as “yes” or “no.” Evaluation of multiples of normal in spleen and liver size was not feasible; “splenomegaly” received a score of 3 irrespective of size, and “hepatomegaly” was scored as 1.5 points. Growth retardation (based on height, weight, and body mass index measures) was defined as equal to or less than the fifth percentile for age. Asthenia and fatigue were scored by one item because both descriptions use the same word in Hebrew. No ICD-9 code was available for family history of GD.

For constructing discrimination models, 16 available features were used as defined in the GED-C scoring system [12]. These included age, anemia, bleeding, bone issues, dyslipidemia, fatigue, gallstones, gammopathy, growth retardation, hepatomegaly, hyperferritinemia, Jewish ancestry, low bone mineral density, leukopenia, splenomegaly, and thrombocytopenia.

Two models were constructed. In the first, all available data were used, including free-text notes from patient visits (database 1). For the second, data from free-text notes from patient visits were not included (database 2).

### Statistical analyses

GED-C summary scores were reported using the median and interquartile range (IQR). Absolute and relative frequencies were reported for nominal data, with the relationship between GD and controls expressed as odds ratio (OR) and 95% CI. A decision tree model using all 16 available features (as detailed above) was constructed using “training data” (80%) and “test data” (20%), and a random forest model was selected and trained in order to identify the main features that can distinguish GD from non-GD controls. The model performance on the validation dataset was evaluated using receiver operating characteristic (ROC) curve and f1-score as a measure of accuracy. The area under the ROC curve (AUC) results are considered excellent for AUC values between 0.9 and 1.0, good for AUC values between 0.8 and 0.9, fair for AUC values between 0.7 and 0.8, poor for AUC values between 0.6 and 0.7, and failed for AUC values between 0.5 and 0.6 [16]. All statistical analyses were performed using R programming version 1.4.1103, packages dplyr, stringr, tidiverse, lubridate, caret, random forest, and ggplot2.

## Results

Of 346 individuals with a GD diagnosis code identified from the MHS database, 265 were confirmed as patients with GD following the screening of patient records. A total of 3445 control individuals without GD (13 per GD case) were included in the study.

Manual screening of free-text notes for terms defined in the GED-C scoring system revealed a marked difference in the proportions of individuals with symptoms between GD and control groups, with ORs of approximately 10 or above for splenomegaly, growth retardation, and hepatomegaly (Table 1). 

**Fig. 1:**
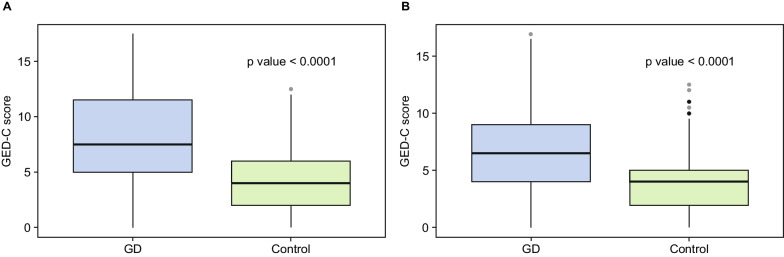
Boxplot of overall GED-C summary scores. **A** Patients with GD versus controls for database 1 (Table 2). **B** Patients with GD versus controls for database 2 (Table 3)

**Fig. 2:**
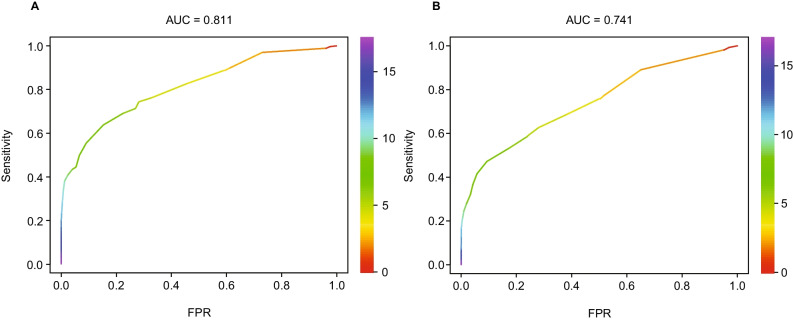
ROC curves for total GED-C scoring. **A** Patients with GD versus controls for database 1 (Table 2). **B** Patients with GD versus controls for database 2 (Table 3). *FPR*, false positive rate

Overall GED-C summary scores were calculated for each database (Fig. 1A, B). For database 1, which included free-text notes from patient visits, median GED-C scores were 7.5 (range 0–17.5; IQR 6.5) for patients with GD (n = 265) and 4.0 (range 0–12.5; IQR 3.5) for controls (n = 3445) (Fig. 1A). GED-C scoring distinguished patients with GD from controls with an AUC of 0.81 (95% CI, 0.78–0.84) (Fig. 2A). For database 2, in which data from free-text notes from patient visits were not included, median GED-C scores were 6.5 (range 0–17.0; IQR 5.0) for patients with GD (n = 265) and 4.0 (range 0–12.5; IQR 3.0) for controls (n = 3445) (Fig. 1B). GED-C scoring distinguished patients with GD from controls with an AUC of 0.74 (95% CI 0.71–0.78) (Fig. 2B).

The magnitude of difference in GED-C scoring between patients with GD and controls was greater for some features (e.g., splenomegaly [OR 80.3 in database 1 and 61.9 in database 2], severe thrombocytopenia [platelet count < 50 × 10^9^/L; OR 57.9 in both databases], and hyperferritinemia [ferritin 300–1000 ng/mL; OR 28.9 in both databases]) (Tables 2 and 3).

**Fig. 3:**
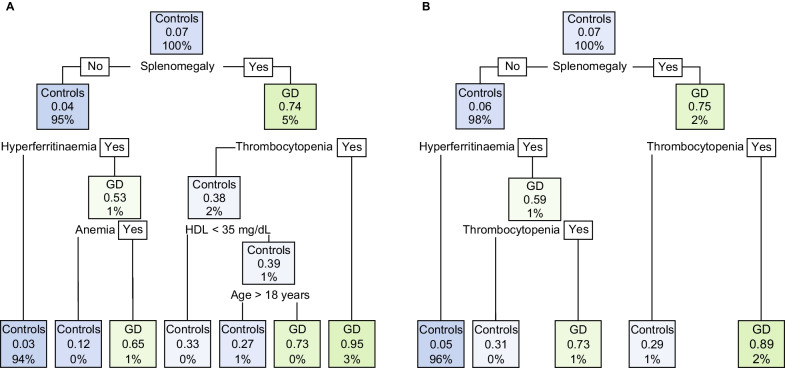
Decision tree models for **A** database 1 and **B** database 2. Features of GD based on GED-C scoring as defined in Table 2 for database 1 and in Table 3 for database 2. As per GED-C scoring: anemia is defined as hemoglobin < 14.0 g/dL, hyperferritinemia is defined as ferritin > 300 ng/mL, and thrombocytopenia is defined as platelet count < 150 × 10^9^/L. Decimal values for p parameter (range 0–1) used to show percentage of the split. *HDL*, high-density lipoprotein cholesterol

Decision tree models were constructed for each database (Fig. 3). The models showed good accuracy (0.96 [95% CI 0.95–0.97] for database 1; 0.95 [95% CI 0.94–0.96] for database 2), high specificity (0.99 [95% CI 0.99–1] for database 1; 1.0 [95% CI 0.99–1] for database 2), but relatively low sensitivity (0.53 [95% CI 0.46–0.59] for database 1; 0.32 [95% CI 0.25–0.38] for database 2). The AUC was higher for database 1 (0.79 [95% CI 0.73–0.87]) compared with database 2 (0.69 [95% CI 0.60–0.73]). The f1-scores were 0.66 for database 1 and 0.46 for database 2.

**Fig. 4:**
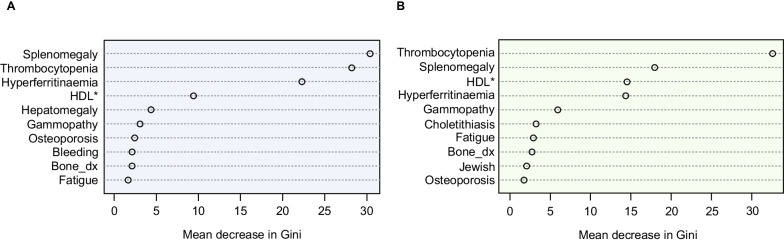
Random forest model showing the top 10 variables for distinguishing patients with GD from controls. **A** database 1 and **B** database 2. Gini impurity describes the probability of incorrect classification at a given node in the decision tree based on training data. Mean decrease in Gini is a measure of a variable’s importance for determining GD across all the decision trees in the random forest: the mean of a variable’s total decrease in node impurity, weighted by the proportion of samples reaching that node in each decision tree in the random forest. *HDL cholesterol < 35 mg/dL. Bone_dx, bone issues, including pain, crises, avascular necrosis, and fractures; *HDL* high-density lipoprotein

A random forest model was developed using the two databases to outline the 10 most important variables for distinguishing patients with GD from controls (Fig. 4). The clinical features of splenomegaly, thrombocytopenia (platelet count < 50 × 10^9^/L), and hyperferritinemia (ferritin 300–1000 ng/mL) were found to be the top three most accurate classifiers of GD in both databases.

## Discussion

This retrospective observational analysis used real-world data to assess the utility of the GED-C scoring system for discriminating between patients with GD and controls. We found that the GED-C scoring system performance at distinguishing between GD and controls was ‘good’ when using a database that included free-text notes from patient visits and ‘fair’ when using a database that excluded free-text notes. Analysis of both databases consistently identified the most important GED-C features for discriminating GD from non-GD as splenomegaly, thrombocytopenia, and hyperferritinemia.

The three features, splenomegaly, thrombocytopenia and hyperferritinemia were also considered by GD experts to be important features in the diagnosis of GD [12]. There are still some differences that need to be discussed. First, the weight given by experts for these three features was significantly lower than the actual odds of exposure found among patients with GD compared with controls in this real-world dataset. Second, GD experts gave a greater weight (2 points) for mild thrombocytopenia (platelet count 50–150 × 10^9^/L) compared with severe thrombocytopenia (platelet count < 50 × 10^9^/L; 1 point), whereas the actual odds of exposure among patients with GD from this study was acutely higher for severe thrombocytopenia (with a wide CI owing to a lower number). For hyperferritinemia, GD experts gave a lower weight for ferritin levels > 1000 ng/mL (1 point), whereas the odds of exposure among patients in this study was approximately 30 for elevated ferritin levels, regardless of the exact levels. Based on our findings, we can expect that a supervised machine learning classifier, that will not use predefined weights or laboratory cutoffs, could perform better in distinguishing between patients with GD and controls.

Although the models developed in this study show high specificity, they were shown to have low sensitivity. Missing or incomplete data for many features may have been a contributing factor. The definitions used in the GED-scoring may further impact the sensitivity of models to discriminate between patients with GD and controls. For example, the definition of anemia using a threshold of 14 g/L for hemoglobin concentration is relevant to adult males, but this may result in the misclassification of normal hemoglobin levels in patients whose hemoglobin would otherwise fall into the anemic range as specified by their age and gender. Modifying the scoring system may help correctly diagnose more patients with GD at an earlier time in the disease course.

As expected, the database that included data from free-text notes (database 1) showed better performance compared with database 2 because physicians frequently documented GD-related features (such as splenomegaly, hepatomegaly, fatigue, and bone pain) in the visit notes but then failed to code them. This is in line with a study of electronic records for splenomegaly in the Danish National Registry of Patients; the total number of patients coded for splenomegaly was lower than expected, leading the authors to conclude that splenomegaly as a clinical finding was probably under-coded in the ICD system [17]. Similarly, machine learning algorithms using ICD-9 codes did not perform well in identifying patients with systemic sclerosis; the highest-performing algorithms in this study incorporated clinical data with ICD, Tenth Revision (ICD-10), codes [18]. Unfortunately, ICD-10 codes were not available in the MHS database. Under-coding could be related to the difficulty in translating signs and symptoms into a clear and unambiguous classification code, coupled with time pressures on physicians [19]. 

The ability of the GED-C to discriminate between individuals with and without GD was also shown by other groups. Mehta et al. compared adults with GD (n = 25) to adults with liver disease, hematological malignancy or immune thrombocytopenia (n = 75) [13]. The data were derived from hospital records, with 80%-90% completeness of data. Analyses, based on 11 possible factors, showed good discrimination between those with GD and non-GD individuals, with AUC of 0.88 (95% CI 0.78–0.97) being comparable to the result from database A in our study. Patients with liver disease and hematological malignancy were most likely to have manifestations overlapping with GD [13]. In a study from Finland, clinical data from five patients with GD were compared to electronic health record data of ~ 170,000 adults from a biobank [14]. The score of patients with GD ranged between 6 and 18.5 (based on the available data on 28–29 possible factors). Only 0.72% of adults from the biobank were assigned at least 6 points, but none had a point score as high as 18.5. A follow-up study using the same approach with another Finnish biobank, showed similar results [20]. The score of 8 patients with GD was 6–22.5 points, while only 0.77% of controls had 10 points or higher. Data from patients with GD collected from hospital electronic medical records being compared with control data collected from a biobank, may explain the significant difference found in the studies from Finland. 

The use of real-world data is both an advantage and a limitation of the study. The use of real-world data confirmed the significance of the majority of features in the GED-C scoring system. These features would need to be included in future machine-learning models. However, real-world data sets have their limitations. They may contain incomplete information or be prone to errors during data entry. In addition, because GD is a rare disease, the number of individuals with GD, even within this relatively large database of individuals, is restricted and there are inherent limitations to the development of an algorithm using such a small data set of patients. In our research, we employed a manual examination of unstructured text notes to identify features related to GD. The reason for resorting to manual screening was that the existing Hebrew resources for training natural language processing (NLP) are inadequate to accomplish this task using computer-based methods [21]. Manual screening was feasible when limited to 265 patients and 265 controls but not for the entire control cohort. Similarly, machine learning diagnostic algorithms will not be able to be based on manual screening of free-text notes. In addition, our study analyzed data from patients retrospectively, a prospective design would have been preferable in terms of standardization of data collection and reducing potential sources of bias.

The current study was set up to extract patient data on parameters from the GED-C score, chosen as a set of readily available clinical data points established by expert clinician consensus to have diagnostic relevance for patients with GD, and also validated in different geographic patient cohorts [13, 14]. However, we acknowledge that findings on the diagnostic utility of this set of variables are limited to the patient population studied and may not be applicable for populations with different ethnic backgrounds and disease severity. Other clinical manifestations of GD not included in the GED-C scoring system should be considered in future algorithms developed for GD diagnosis, for example co-morbidities that may be associated with GD such as neoplasms, endocrinological disorders like insulin-like growth hormone deficiency, as well as abnormalities in parathyroid hormone levels, phospho-calcium metabolism, and vitamin D levels [22–25]. 

In the era of digitized medical records, the opportunity for comparing and combining electronic health data from a wide variety of patient populations is likely to result in better refinement of data-mining tools. As seen with other diseases, use of machine learning for analysis of large amounts of clinical data will assist the development of algorithms for detecting undiagnosed patients with GD, as well as tools optimised for use in particular patient sub-sets [26, 27]. This, accompanied by large-scale testing of biobank samples using alternative diagnostic techniques, is needed to address the challenges of GD diagnosis in the future.

**Fig. 5:**
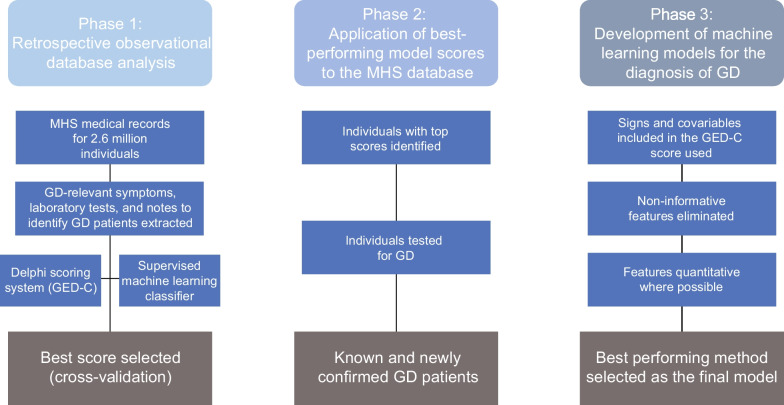
Study design for the development of an artificial intelligence–based algorithm. *GD* Gaucher disease, *GED-C* Gaucher early diagnosis consensus, *MHS* Maccabi Health Services

This study forms part of the basis for the development of an artificial intelligence–based algorithm for the accurate diagnosis of GD using machine learning, designed to shorten the diagnostic journey of patients with GD (Fig. 5). Machine learning technologies are being developed for a number of disorders that can potentially accelerate accurate diagnosis by calculating disease probabilities based on symptoms [28, 29]. Integration of machine learning models employing quantifiable variables that are readily discernible and autonomous of clinical evaluations to facilitate screening for rare diseases, such as GD, are needed to improve early detection and treatment [30]. In the next phase of the research process, the models evaluated in this study are to be further refined, by incorporating additional parameters and using supervised machine-learning methods. The best-performing model would then be applied to the MHS healthcare database population to identify those who may have unidentified GD. These data would then be used for further refinement of the machine learning classifiers for GD diagnosis.

## Conclusion

The GED-C score, developed by Delphi expert consensus, shows good discrimination between patients with GD and controls and could be the basis for future models. In our study, as expected, the database that included data from physician notes on frequently documented GD-related features showed the best performance at distinguishing GD patients from controls. The application of machine learning techniques to our cohort is expected to result in an improved diagnostic model with the best possible sensitivity that may be used for screening undiagnosed GD cases.

## Data Availability

The data that support the findings of this study are available from the authors but restrictions apply to the availability of these data, which were used under license from the Maccabi Health Services (Israel) for the current study, and so are not publicly available. Data are, however, available from the authors upon reasonable request and with permission from MaccabiTech, Maccabi Health Services, research center.

## Abbreviations

AUC: Area under the receiver operating characteristic curve
GD: Gaucher disease
GED-C: Gaucher earlier diagnosis consensus
ICD-9/10: International classification of diseases, ninth/tenth revision
IQR: Interquartile range
MHS: Maccabi Healthcare Services
OR: Odds ratio
ROC: Receiver operating characteristic
